# Aldosterone up-regulates voltage-gated potassium currents and NKCC1 protein membrane fractions

**DOI:** 10.1038/s41598-020-72450-4

**Published:** 2020-09-24

**Authors:** Parveen Bazard, Bo Ding, Harish K. Chittam, Xiaoxia Zhu, Thomas A. Parks, Thomas E. Taylor-Clark, Venkat R. Bhethanabotla, Robert D. Frisina, Joseph P. Walton

**Affiliations:** 1 Department of Medical Engineering, College of Engineering, University of South Florida, Tampa, FL 33620 USA; 2 Department Communication Sciences and Disorders, College of Behavioral and Communication Sciences, Tampa, FL 33620 USA; 3 Department of Chemical Engineering, College of Engineering, University of South Florida, Tampa, FL 33620 USA; 4 Global Center for Hearing and Speech Research, University of South Florida, Tampa, FL 33612 USA; 5 Department of Molecular Pharmacology and Physiology, University of South Florida, Tampa, FL 33620 USA

**Keywords:** Physiology, Molecular medicine

## Abstract

Na^+^–K^+^–2Cl^−^ Cotransporter (NKCC1) is a protein that aids in the active transport of sodium, potassium, and chloride ions across cell membranes. It has been shown that long-term systemic treatment with aldosterone (ALD) can enhance NKCC1 protein expression and activity in the aging cochlea resulting in improved hearing. In the present work, we used a cell line with confirmed NKCC1 expression to demonstrate that in vitro application of ALD increased outward voltage-gated potassium currents significantly, and simultaneously upregulated whole lysate and membrane portion NKCC1 protein expression. These ALD-induced changes were blocked by applying the mineralocorticoid receptor antagonist eplerenone. However, application of the NKCC1 inhibitor bumetanide or the potassium channel antagonist Tetraethyl ammonium had no effect. In addition, NKKC1 mRNA levels remained stable, indicating that ALD modulates NKCC1 protein expression via the activation of mineralocorticoid receptors and post-transcriptional modifications. Further, in vitro electrophysiology experiments, with ALD in the presence of NKCC1, K^+^ channel and mineralocorticoid receptor inhibitors, revealed interactions between NKCC1 and outward K^+^ channels, mediated by a mineralocorticoid receptor-ALD complex. These results provide evidence of the therapeutic potential of ALD for the prevention/treatment of inner ear disorders such as age-related hearing loss.

## Introduction

Aldosterone (ALD) is a primary mineralocorticoid hormone, specifically, a steroid hormone produced by the zona glomerulosa of the adrenal cortex of the adrenal gland^1^. A well-known role of ALD is to promote trans-epithelial Na^+^ and K^+^ transport in the kidney, thereby regulating blood volume and pressure. ALD also targets other organs, such as heart^2^, intestine^3^ and cochlea^4^. ALD binds with mineralocorticoid receptors in the cytosol resulting in an ALD-receptor complex that initiates the transcription of an ALD regulated gene—SGK1. This mediates modification of different ion channel functions and activities including sodium, calcium, magnesium and potassium channels, as well as calcium activated potassium channels^5–11^. For instance, SGK1 upregulates the function of apical epithelial Na^+^ channels in kidney^5,6,9^. SGK1 also modulates functionality and expression levels of renal outer medullary K^+^ channels, which are apically located secretory channels in the distal nephron^12,13^. Other reports indicate that Ca^2+^ and Mg^2+^ channels can be modulated by ALD-sensitive SGK1 gene^11,14^.

In addition to SGK1, there are other transcriptionally upregulated proteins like glucocorticoid-induced leucine zipper proteins, and scaffold protein connector enhancer of kinase suppressor of Ras isoform 3 (CNK3) as well, which mediate the ion expression and activity levels^15,16^**.** Pertaining to ALD sensitive channel currents involving NKCC1, it has been reported that ALD induced an increase in Na^+^–K^+^ pump currents and Na^+^ influx in rabbit ventricle cardiomyocytes^17^. This increase was sensitive to bumetanide, indicating that activation of NKCC1 by ALD led to an increase in Na^+^ influx. Similarly, it has been shown that ALD can modulate Na^+^ absorption as well as Cl^−^ secretion in hen colon, which was sensitive to the NKCC1 blocker—bumetanide^18^.

Bumetanide is also a diuretic which has a 500-fold greater affinity to NKCC1, compare to potassium-chloride transport member 5 (KCC2) and is an ideal candidate to study NKCC1 physiological actions^4,19,20^. Na^+^ K^+^ 2Cl^−^ cotransporter (NKCC1) is ubiquitous throughout the body that regulates transport of Na^+^, K^+^ and Cl^−^ ions across the cell membranes. NKCC1 transports ions into and out of cells, driven energetically across the membrane because of gradients between Na^+^ and/or K^+^ and Cl^−^ ions^21,22^. Inside cells, NKCC1 is commonly found in the basolateral membrane^23^, the part of the cell membrane which is oriented away from the lumen of the tubule and closest to blood vessels. This unique cellular location provides NKCC1 the ability to transport sodium, potassium, and chloride from the blood into cells.

In addition to exocrine glands, NKCC1 plays a critical role in establishing the potassium-rich endolymph in the inner ear that is essential for normal hearing. Age-linked stria vascularis degeneration and dysfunction have been implicated as a primary cause of age-related hearing loss (ARHL), affecting hundreds of millions worldwide and is sometimes referred to as strial presbycusis^24–33^. Cells within the stria vascularis (SV) are responsible for maintaining the high ionic concentration of K^+^ potassium which generates the endocochlear potential (EP), a biological battery required for cochlear function and normal hearing^34–36^. When the NKCC1 antagonist furosemide is applied to the cochlea the EP decreases in magnitude and also elevates the thresholds of auditory nerve fibers^35,37^. EP declines with age are referred to as metabolic presbycusis, involving the pathogenesis of age-related SV degeneration^38^. Specifically, in strial marginal cells, NKCC1 is essential for the production of a potassium-rich endolymphatic fluid^4,23,39^.

ALD, as a naturally occurring hormonal agonist for NKCC1, plays an important role in the maintenance of Na^+^, K^+^ and acid–base balance in inner ear^4,40^. Our recent work has shown that long-term systemic treatment with ALD slows down the progression of ARHL in aging mice, probably by increasing SV Na^+^–K^+^ transport and up-regulation of NKCC1 protein expression mediated by mineralocorticoid receptors^4,39–41^. The voltage gated K^+^ ion channels encoded by KCNQ4, Kv1.1, Kv 3.1 and Kv 4.2 are expressed in various parts of the peripheral and central auditory systems; including the outer hair cells and cochlear nucleus, and plays a significant role in potassium circulation in the inner ear^29,42–45^. However, it is not clear whether changes in NKCC1 protein expression and activity in response to changing levels of ALD parallel changes in voltage-gated K^+^ channel currents. This unanswered question provided the motivation for this study to investigate the biophysical mechanisms by which ALD modulates NKCC1 channel function. For the present study, whole-cell patch clamp recordings were used to study the single-cell physiological changes involving NKCC1 following application of ALD and its antagonists in a neuronal cell line. Our goals were (1) Determine NKCC1 expression variants by stimulating SH-SY5Y neural cells with ALD and (2) Explore characteristics of NKCC1 functionality by recording voltage-gated K^+^ outward currents driven by ALD.

## Methods

### Cell culture

SH-SY5Y (ATCC® CRL-2266™) neuroblastoma cells were initially cultured in a medium containing a mixture of F12 & DMEM (1:1 volume/volume v/v) supplemented with 1% Penicillin Streptomycin (Pen Strep) and 10% FBS. The cells were then incubated in an incubator maintained at 37^o^ C temperature, 5% CO_2_ and 95% humidity. The medium was replaced after every 4–7 days. After 80–90% confluence, cells were incubated with trypsin solution for 1–2 min to detach adherent cells. Cells with trypsin solution were combined with equal volume of F12 & DMEM (1:1 v/v) medium with 1% Pen Strep and 10% FBS to neutralize the trypsin. The resultant solution was then centrifuged at 1,500 rpm for 5 min. To subculture the cells, the pellets were then suspended in F12 & DMEM (1:1 v/v) medium with 1% Pen Strep and 10% FBS^46–48^. SH-SY5Y cell lines can be differentiated to neurons. The differentiation of SH-SY5Y cells is achieved by adding retinoic acid (RA) to the cell culture medium. RA has known to possess cellular differentiation and growth inhibiting properties^49–51^. Within 48 h of plating, the serum-containing medium was replaced with neurobasal medium supplemented with B27 (serum-free supplement, #17504044, Life Technologies, Carlsbad, CA) and GlutaMAX. To promote differentiation, 10–20 µM all-trans-retinoic acid was added to the cell culture medium and the medium was replaced after every 48 h. Differentiated cells were observed under the microscopy and were more pyramidal shaped, distributed, and had extended neurites^47^.

### Cell Fraction Isolation

A subcellular protein fractionation kit (#9038, Cell Signaling Technology, Danvers, MA) was used to separate cellular proteins into nuclear, membrane and cytoplasmic fractions following the manufacturer’s protocol. This is achieved by using different detergents that take advantage of the inherent structural and composition features of different cellular fractions^52–55^. *Isolating cell population:* The cultured cells were washed with cold 1X PBS and detached from the surface using trypsin. 100 µl of cell suspensions aliquot was used for the whole cell lysate (WCL). 60 µl of 3X SDS Loading Buffer with DTT (#7722, Cell Signaling Technology, Danvers, MA) was added to make a final volume 160 µl for WCL. WCL tube was sonicated for 15 s at 20% power 3 times, heated for 5 min at 95 °C, and centrifuge for 3 min at 15,000×*g*. *Fractions Isolation:* The remaining 400 µl lysate was transferred into a 1.5 ml tube and centrifuged for 5 min at 500 × g at 4 °C. The supernatant was aspirated. The pellet was suspended in 500 μl of cytoplasm isolation buffer (CIB), vortexed for 5 s, followed by incubation on ice for 5 min and centrifugation for 5 min at 500×*g*. The supernatant is the cytoplasmic fraction and this pellet was resuspended in 500 μl of membrane isolation fraction (MIB) and vortexed for 15 s. Then, it was incubated on ice for 5 min and was centrifuged for 5 min at 8,000×*g*. The supernatant was the membrane fraction. These fractions were analyzed by SDS-PAGE and western blotting.

### RT-PCR experiments

SH-SY5Y cells total RNA was extracted according to RNAeasy Mini Kit (Qiagen Inc., Valencia, CA) instructions. The RT-PCR analysis was performed as previously described^4,56^. In brief, total samples were vortexed for 1 min to shear genomic DNA before loading onto the RNeasy mini columns, and then eluted in a minimum volume of 30 µl and a maximum volume of 2 × 50 µl RNAse-free water. RNA obtained with this procedure was essentially free of genomic DNA. 50 ng of RNA was reverse transcribed and complementary DNA was subjected to PCR amplification. Quantitative reverse-transcription polymerase chain reaction (qRT-PCR) was performed using the Enhanced Avian HS RT-PCR-100 Kit (HSRT20, Sigma, St. Louis, MO). The reverse transcript (RT) reaction took place at 46 °C for 20 min. The competition between primer sets was excluded by adjusting the reaction condition. Then, RT products went to PCR amplification directly. Thermal cycle protocol was 2 min at 94 °C, followed by 15 s at 94 °C, 30 s at 55 °C and 1 min at 68 °C for 25 cycles and final holding for 5 min at 68 °C. The conditions were chosen so that none of the RNAs analyzed reached a plateau at the end of the amplification protocol, i.e. they were in the exponential phase of amplification. Each set of reactions always included a no-sample negative control. We also performed a negative control containing RNA instead of cDNA to rule out genomic DNA contamination. The PCR products were analyzed on agarose gels stained with Gel Red Nucleic Acid Stain (Biotium, Hayward, CA).

The quantitative real-time RT-PCR reaction mixture was prepared using the EvaGreen PCR master mix. Thermal cycling conditions were the same as in the semi-quantitative method. Amplification specificity was checked using melting curves. Both negative and positive controls were included in each PCR reaction. All assays were carried out three times as independent PCR runs for each cDNA sample. Gene expression was referenced to the expression of β-actin as the housekeeping gene. Each gene expression level was normalized with respect to β-actin mRNA content. Calculations of expression were performed with relative standard curve method^57^. The primers were as follows: NKCC1- CACTGGAGAGCAAGAAGCCA (F), GGCTGACTGAGGATCTGCAA (R) [Amplicon Size—185 bp] and β-actin-AGCTGTGCTACATCCACGAA (F), AATGCCAGGGTACATGGTGG (R) [Amplicon Size—118 bp].

### Electrophysiology

The electrophysiology experiments were performed using a standard whole-cell in vitro patch clamp technique, utilizing a 700 B Multiclamp amplifier and 1,440 A data acquisition unit from Molecular Devices (Sunnyvale, CA). Micropipettes were pulled from borosilicate glass tubing (Sutter Instrument, Novato, CA) with resistances in the range of 2–4 MΩ. The recordings were performed in the whole-cell configuration at room temperature. Once giga-seal was achieved, slight negative pressure was applied to achieve the whole-cell configuration. The extracellular recording solution contained the following (in mM): NaCl 125; KCl 4; CaCl_2_ 2; MgSO_4_ 1.2; Glucose 10; 0.3 µM TTX; HEPES ( 4-(2-Hydroxyethyl) piperazine-1-ethanesulfonic acid, N-(2-Hydroxyethyl) piperazine-N′-(2-ethanesulfonic acid)) 10, and the pH was adjusted to 7.4 with NaOH. This solution was continuously perfused at the rate of approximately 1 ml/min. The NKCC1 affecters (bumetanide, ALD) were added to the extracellular solution. The intracellular electrode solution contained the following components (in mM): KCl 140; NaCl 4; CaCl_2_ 0.02; EGTA (Ethylene glycol-bis(2-aminoethylether)-*N,N,N′,N′*-tetraacetic acid) 0.8; MgCl_2_ 2; MgATP 4; HEPES 10; and the pH was adjusted to 7.4 with KOH^46,58^. In voltage clamp mode, currents were recorded for a range of − 60 mV to + 30 mV testing potentials at increments of 10 mV, with a holding potential of − 90 mV. No series compensation was performed; however, we did calculate the error due to the change in series resistance during a recording and it was under 5% for all the recordings. The variation in time constant is representative of series resistance errors, given that whole cell capacitance does not change during a recording. Table 1 shows representative calculations for one representative trace. This error was substantially lower than the magnitude of the whole cell response following the applications of various drugs. Clampex10.3 (Molecular Devices, Sunnyvale, CA) software was used for recording and acquisition, and the Multiclamp software was used for stimulation. Whole-cell currents were sampled at 10 kHz with an interval of 100 µs and low pass filtered at 1 kHz with a Gaussian type filter ^47^.

**Table 1:** Time constant calculations for a representative trace.

S. no	Testing voltage (mV)	Time constant (ms)
1	−40	0.223
2	−30	0.230
3	−20	0.235
4	−10	0.231
5	0	0.232
6	10	0.222
7	20	0.223
8	30	0.215

Holding potential: − 90 mV.

### Western blot analysis

The Western Blot analysis was performed as previously reported^4^. In brief, cell (scraping) or tissue (homogenized) lysates were prepared in radio-immunoprecipitation assay buffer (RIPA, Pierce #89901, Thermo Scientific, Waltham MA) with protease inhibitor cocktail (#78430, Thermo Scientific, Rockford, IL). Cell samples were homogenized in buffer, followed by centrifugation at 2000× for 10 min at 4 °C. Supernatants were subjected to western blot analysis by loading 20 μg protein per lane, after the protein concentrations were determined by the Bradford protein assay. Proteins were fractionated by SDS-PAGE gel electrophoresis and transferred to a PVDF blotting membrane. The blot was incubated with primary antibodies and subsequently with the secondary antibody. First antibody dilution was 1:1000 and the second antibody was 1: 2000. The primary antibodies for NKCC1 and β-actin were monoclonal rabbit IgG (D13A9, #8351 Cell Signaling Technology, Danvers, MA) and polyclonal rabbit (#4967, Cell Signaling Technology, Danvers, MA). The secondary antibody was goat ant-rabbit IgG HRP linked (#4967, Cell Signaling Technology, Danvers, MA). In addition, the pre-experiments showed that target protein expression was proportional to housekeeping gene expression levels (β-actin).

### Statistical analyses

Images from Analytic Jena UVP Chem Studio SA2 were imported to NIH Imaging J software for the densitometry analysis and the data reported as mean ± SD. Statistical analysis was performed with GraphPad Prism 5.0 (GraphPad, La Jolla, CA). Differences were analyzed with a 1-way or a 2-way repeated measure analysis of variance (ANOVA) as appropriate, or a two-way ANOVA followed by Bonferroni post hoc t-tests and corrected for multiple comparisons with *p* < 0.05 considered significant.

## Results

### NKCC1 expression in SH-SY5Y cells

**Figure 1:**
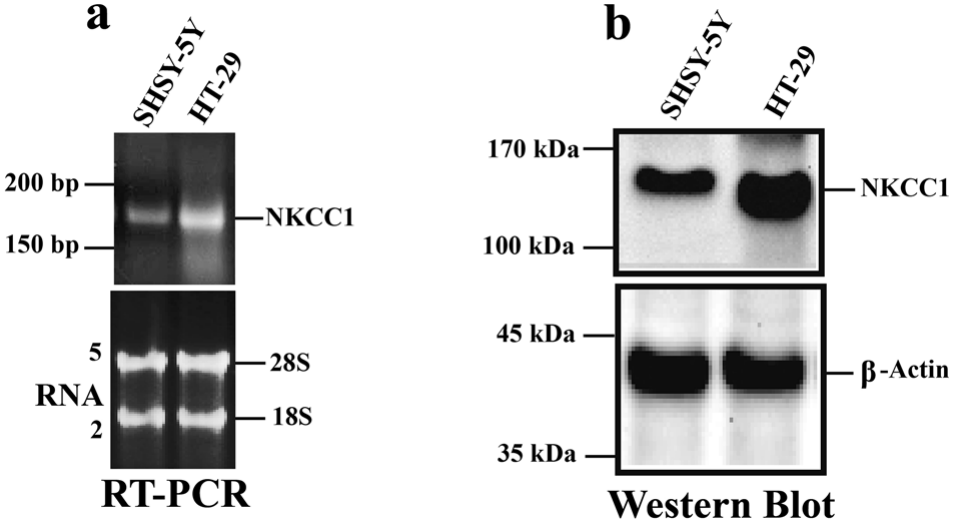
NKCC1 genes and proteins are expressed in SH-SY5Y neural cells. Gene and protein expressions were analyzed using RT-PCR and western blotting techniques, respectively. (**a**) RNA was isolated from SH-SY5Y cells and was reverse transcribed, then the cDNA from RT was used for real time PCR (qPCR) to quantify RNA levels. Single band on the right side is HT-29 control cells in both panels a and b. NKCC1 gene expression was detected between 150–200 bp size, for SH-SY5Y cells (**a**, Top). The bottom lanes show that total RNA was run and the clear 18S and 28S ribosomal RNA bands show that the RNA was intact with no degradation. Total RNA was also used as the loading control for both SH-SY5Y cells and HT-29 Cells and for the evaluation of NKCC1 gene expression difference between SH-SY5Y and HT-29 cell lines (**b**) Cell lysate was collected in RIPA buffer and analyzed using western blots for NKCC1 protein expression. Data indicate that both SH-SY5Y and HT-29, control cells show NKCC1 expression. For western blot experiments, both proteins of interest, NKCC1 and the loading control protein, β-Actin, were probed on the same PVDF membrane i.e., same membrane were incubated with NKCC1 and β-Actin antibodies. Similarly, for semi-quantitative RT-PCR, both the gene of interest (NKCC1) and reference gene (β-Actin) were probed together using respective primers and analyzed on the same agarose gel.

To determine whether NKCC1 was expressed in SH-SY5Y neural cells, we selected primers designed to span key introns and bridge an exon-to-exon junction for RT-PCR assays. HT-29 cells were used as a positive control because these cells have abundant NKCC1 expression^4,59^. The NKCC1 gene was detected at 150–200 bp (Fig. 1a upper panel). Total RNA quality was confirmed before the process of reverse-transcription (RT) reaction using electrophoresis (Fig. 1a—lower panel). The presence of 18 s and 28 s ribosomal bands showed that the RNA was intact and not degraded and, indicates a valid loading control for both cell lines—SH-SY5Y cells and HT-29 (positive control). NKCC1 protein expression was observed at approximately 160 kDa in the SH-SY5Y cells (Fig. 1b), and the positive control NKCC1 expression in HT-29 cells is shown in the farthest right lane.

### Effect of aldosterone on NKCC1 expression levels and whole-cell voltage-gated potassium currents

**Figure 2:**
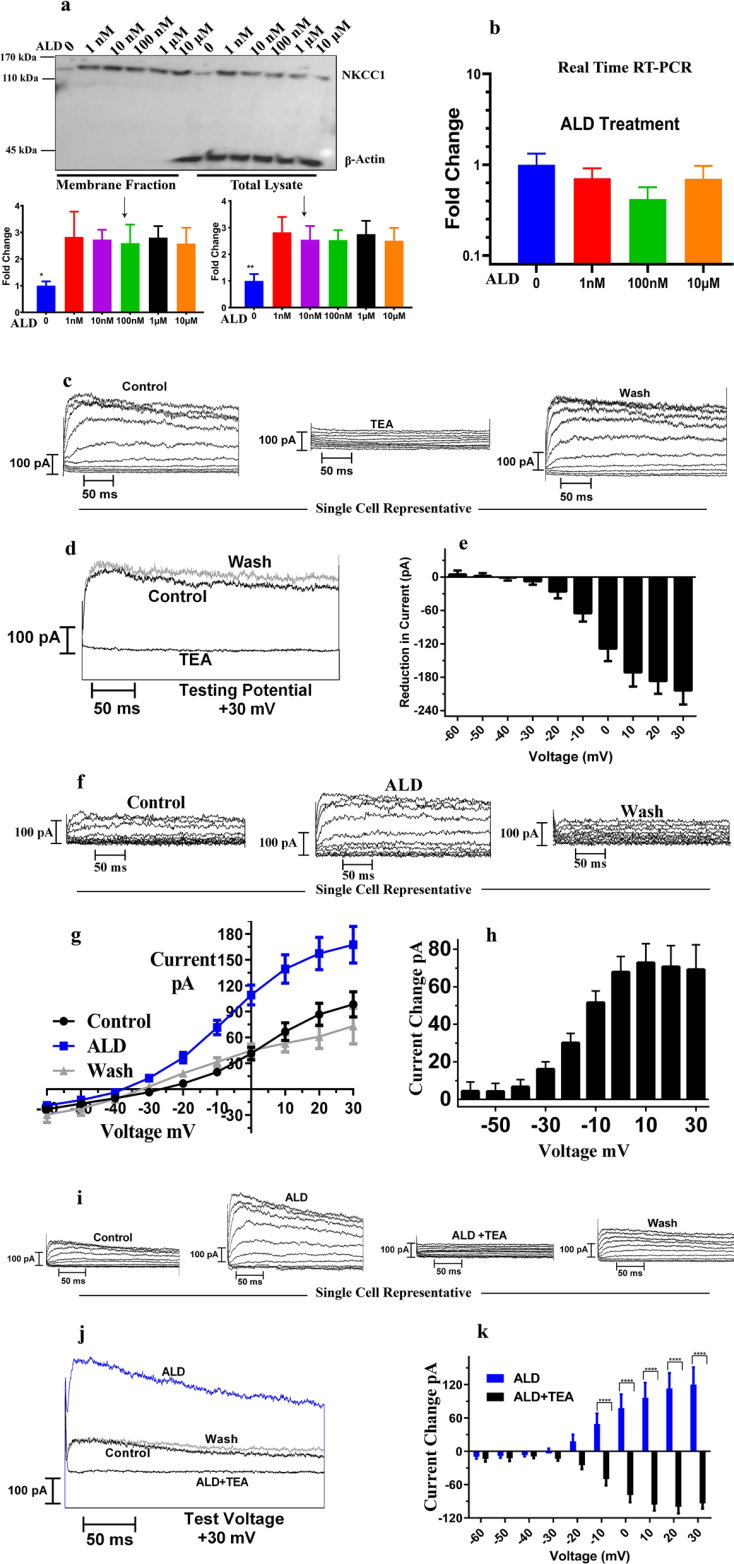
Aldosterone induces increases in NKCC1 protein expression levels and outward potassium current amplitudes. (**a**) SH-SY5Y cells were treated with aldosterone (ALD) at concentrations from 1 nM to 10 μM for 24 h. Cell lysates were performed in PBS buffer and membrane fractions were separated using the cell signaling kit. Western blot analysis probed with NKCC1 and β-actin antibodies for both total lysate and membrane fractions. β-actin was used as the loading control and equal amounts of lysate protein were used for the isolation of cell membrane proteins (lysate protein concentration was measured by spectrophotometer). For both total lysate and membrane fractions, NKCC1 protein expression was upregulated following application of ALD as compared to control samples (no ALD treatment). There was no β-actin present in the membrane fractions as it is a structural protein. Data are means ± SD from 3 independent experiments. (**b**) NKCC1 mRNA was not regulated by ALD treatment. Cell lysates performed in RLT buffer and total RNA was isolated using the Qiagen kit. Subsequently, real-time RT-PCR technique was carried out. There was no significant difference between the control (no ALD) and treated samples (n = 3). All western blot data were taken from the same gel. (**c**) Whole cell voltage-clamp recordings of a representative SH-SY5Y show outward currents elicited by 300 ms voltage steps. Voltage steps from -60 mV to + 30 mV, in 10 mV step increments were acquired at a holding potential was −90 mV. There was a reversible decrease in K^+^ currents due to application of tetraethyl ammonium – TEA (1 mM), confirming the presence of K^+^ channels in SH-SY5Y cells. (**d)** For a representative cell, current traces at + 30 mV voltage step shows the effects of TEA on K + currents. (**e**) Similarly, mean reduction in currents with respect to control (n = 6) was shown when cells were perfused with 1 mM TEA. (**f)** There was a reversible increase in potassium currents when 1 μM ALD was applied as compared to no ALD and currents decreased to the control values when ALD was washed out. (**g)** Mean I–V functions are plotted for control (n = 17 cells) and after ALD application (n = 5 for wash) were shown as mean current–voltage relations. (**h)** The change in outward currents with respect to control conditions following ALD application were greater for more positive holding potentials. (**i)** To further confirm the increase in K^+^ due to ALD application, ALD was perfused in combination with the K^+^ blocker, TEA. Currents traces of a representative cell were shown as outward currents in response to test potentials −60 mV to + 30 mV, in 10 mV increments with holding potential −90 mV. There was an increase in potassium currents due to ALD, followed by decrease in currents when TEA was added to the bath. Currents return to control values during wash out. (**j, k**) Current traces at + 30 mV testing voltage and mean change with respect to control values in currents confirm the same tendency (n = 5). Statistical Significance: *P < 0.05, ** P < 0.01, ***P < 0.001, ****P < 0.0001.

Our previous study indicated that ALD can directly control expression of NKCC1 in HT-29 cells^4^. To confirm a similar result in SH-SY5Y cells, we measured NKCC1 protein and mRNA expression following application of ALD as shown in Fig. 2a. Functional expression of NKCC1 in SH-SY5Y cells showed that there is an increase in both the total cell lysate amount of NKCC1 and the cell membrane fraction, which remained relatively stable across a large range of doses (1 nm–10 µm). The threshold of NKCC1 induction was quite sensitive, as there was a significant response even at 1 nM. β-actin is a structural protein, used as a loading control but is not located in the cell membrane, so there was no β-actin expression in the membrane portion (Fig. 2a). Total lysate concentration values were used to achieve equal protein amounts for membrane fraction processing.

We also measured the effects of ALD on NKCC1 mRNA levels using semi-quantitative and real time RT-PCR methods to determine if NKCC1 protein expression increases were associated with RNA-level induction changes. Both methods indicated that there was no difference in mRNA expression between treated and control samples (Fig. 2b), revealing that the ALD-induced increase of NKCC1 protein expression is associated with post-transcriptional modification.

To determine whether ALD affects whole-cell voltage-gated K^+^ currents, in vitro electrophysiology experiments were performed. Voltage-gated potassium channels (Kv), encoded by 40 genes in humans, are the largest subset of potassium channels that are gated by changes in cell membrane potential^60^. In addition, both ALD and NKCC1 regulate Na^+^ and K^+^ transport for cells, so we hypothesized that ALD could mediate voltage-gated potassium currents associated with NKCC1 modulation. In vitro electrophysiology experiments were done in whole cell voltage clamp mode. First, experiments were done to confirm the presence of tetraethyl ammonium (TEA—1 mM) sensitive outward K^+^ currents in SH-SY-SY5Y cells. As shown in Fig. 2c, we observed a decrease in K^+^ current magnitudes when cells were perfused with TEA, and that decrease was reversible following washout with an extracellular solution. Representative currents of a cell at testing voltage + 30 mV and mean reduction in currents (n = 6) confirms the same result: the presence of outward K^+^ currents in SH-SY5Y cells (Fig. 2d, e). ALD was added to the extracellular solution and was continuously perfused. An increase in the outward current was observed when ALD was perfused for approximately 10 min, as presented in Fig. 2f. Increases in outward currents were reversible and currents returned to pre-application values when washed with extracellular solution. Mean I-V curves confirmed the same trend as shown in Fig. 2g. ALD treatment resulted in an increase in outward currents (blue curve—n = 17) as compared to perfusion with extracellular solution having no ALD (black curve—n = 17). This increase in outward currents was completely reversible following a washout (gray, n = 5). The shift in current from baseline was more prominent for positive holding potentials (0–30 mV) as shown in Fig. 2h. For additional confirmation that ALD up-regulates K^+^ current cells were perfused with ALD and ALD in combination with the K^+^ blocker TEA. Consistent with results presented above, there was increase in K^+^ currents, followed by a decrease in currents when cells were perfused with ALD & TEA. K^+^ current magnitudes went back to control values when cells were washed with extracellular solution (Fig. 2i). Figure 2j shows the current traces at a testing voltage + 30 mV, with a holding potential of – 90 mV, showing similar results. The changes in current magnitudes (increase due to ALD and decrease due to ALD + TEA) was more prominent for positive testing voltages.

### NKCC1 membrane protein upregulation by aldosterone is regulated via mineralocorticoid receptors

**Figure 3:**
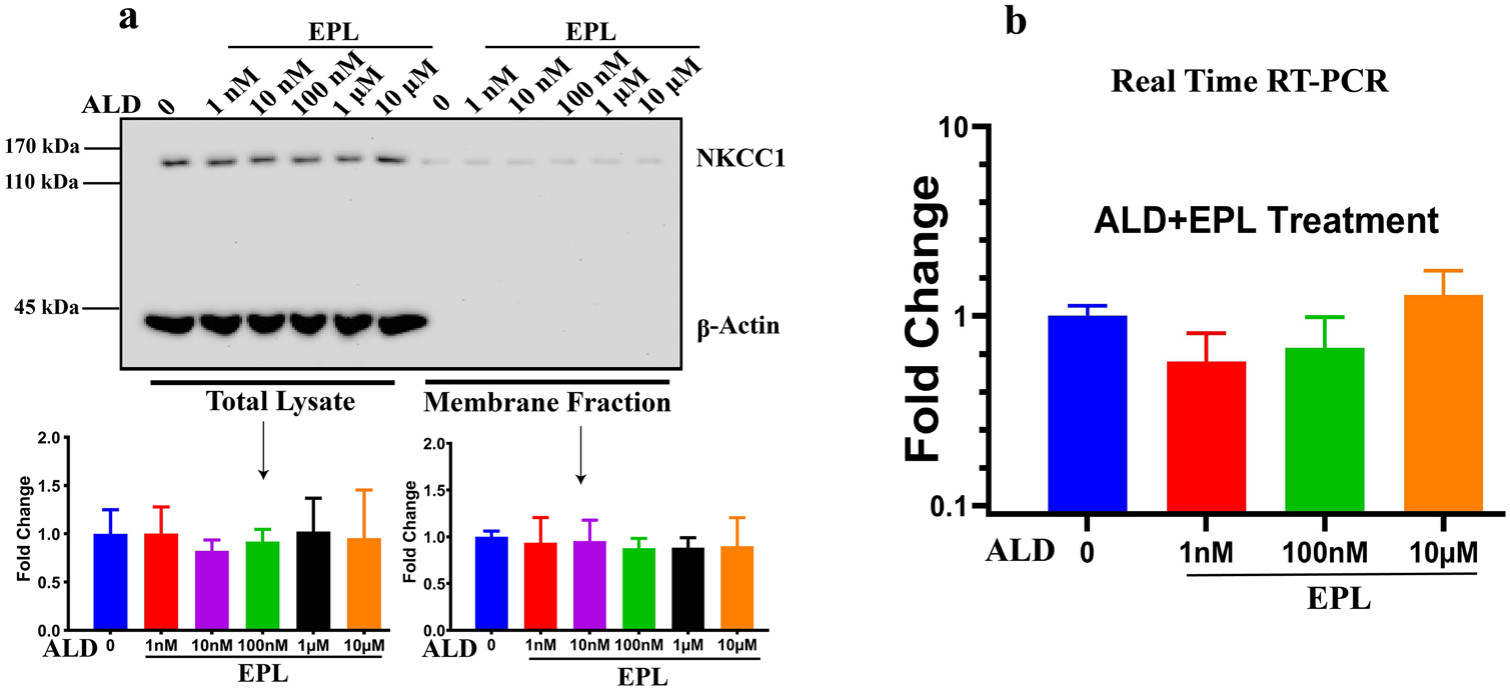
NKCC1 protein levels were increased by upregulation of mineralocorticoid receptors. (**a**) Cells were treated with eplerenone (EPL—20 µM), a specific mineralocorticoid receptor inhibitor. Cell lysates were collected in PBS buffer and analyzed for total lysate and membrane fraction for NKCC1 protein expressions using western blot techniques. Equal amounts of lysate protein were used for the isolation of cell membrane proteins (according to the lysate protein concentration measured by spectrophotometer). β-actin was used to achieve equal loading for total lysate, but β-actin is not found in membrane fractions. The blue bar is the control, untreated cells and remaining are various colors with concentration of ALD from 1 nM to 10 µM along with EPL. a) There was no difference between control and treated samples (ALD + EPL) i.e., ALD did not upregulate NKCC1 protein expression in the presence of inhibitor. Bar graphs show mean ± SD from 3 independent runs. (**b**) Similar to protein expression, there was also no difference in gene expression for NKCC1 following ALD treatment in the presence of eplerenone from 1 nM to 10 µM. Bar graph data are presented as mean ± SEM for three independent runs.

Steroid-hormone actions involve initial binding to intracellular receptors, then binding of this hormone–receptor complex to DNA and activation or repression of target gene transcription. However, steroid hormones can also act through non-genomic mechanisms and thereby change physiological processes^61^. In contrast to genomic steroid actions, these non-genomic effects are characterized by rapid onset and insensitivity to transcriptional and translational inhibitors. We observed similar rapid action of ALD in our experiments and we then determined whether ALD exerts its effect on NKCC1 through the activation of mineralocorticoid receptors. Cells were treated with ALD and eplerenone (EPL—20 μM), a selective potent ALD mineralocorticoid receptors antagonist. Total lysate and membrane fractions were analyzed using western blots. As shown in Fig. 3a, EPL prevented upregulation of NKCC1 protein expression by ALD for both total lysate and membrane fractions. β-actin was used as the loading control and as expected the β-actin signal was not observed in membrane fractions; therefore, total lysate concentrations were used to achieve equal loading for the isolation of membrane fractions. Similarly, there was no change in mRNA levels due to treatment—ALD + EPL (Fig. 3b). These results indicate that modulation of NKCC1 protein levels and the induction of membrane proteins by ALD were mediated by the activation of mineralocorticoid receptors. This inhibition of NKCC1 protein upregulation by ALD with EPL is not tied to mRNA changes, indicating that the ALD-mediated mineralocorticoid receptors pathway is not associated with NKCC1 transcriptional activation.

### NKCC1 and potassium channel inhibitors do not affect ALD-induced NKCC1 protein expression

**Figure 4:**
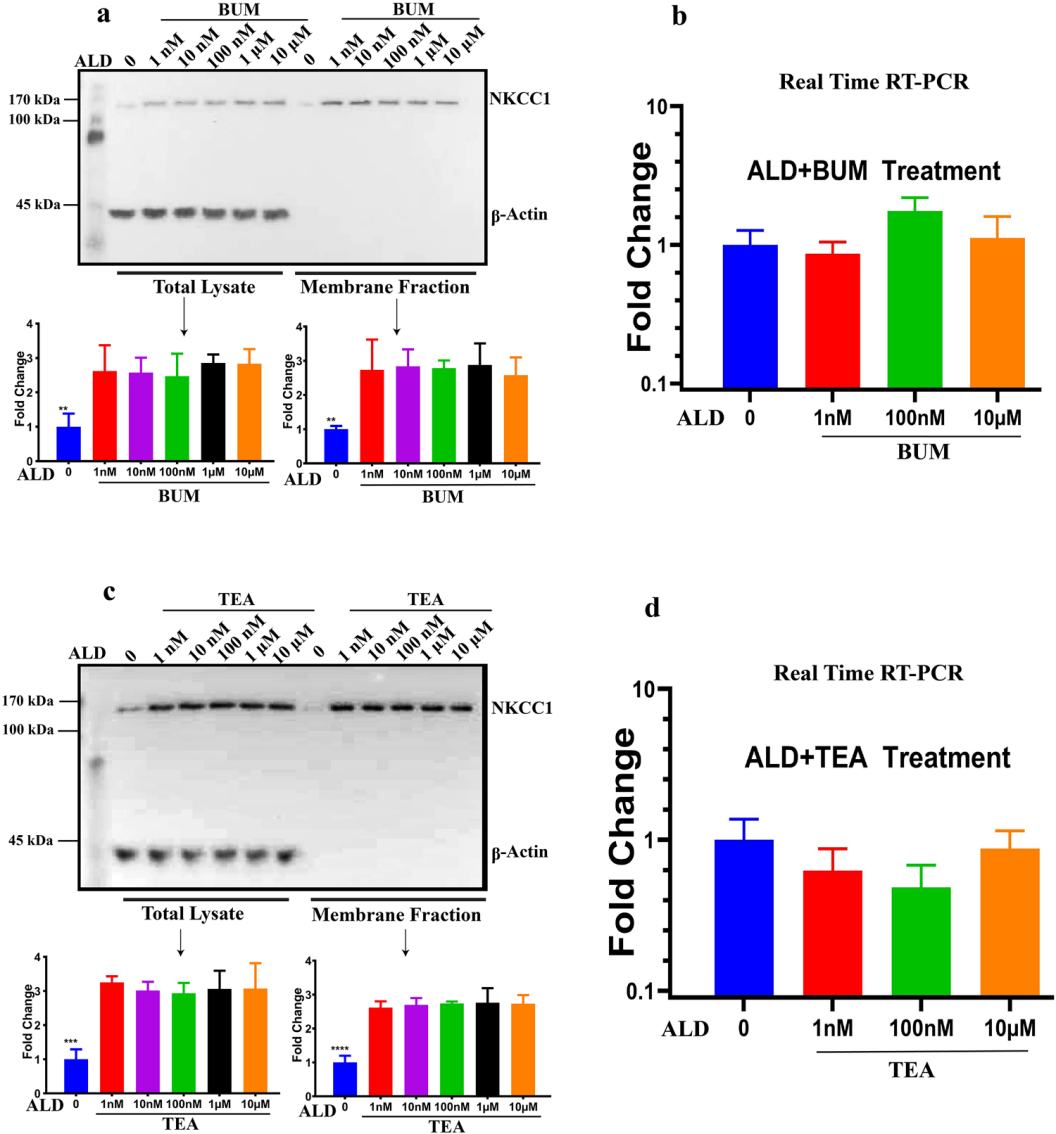
The NKCC1 inhibitor bumetanide, and potassium channel blocker TEA, did not alter ALD mediated changes in NKCC1 protein expression via post-transcriptional modification. (**a**) Cells were treated with ALD in presence bumetanide (BUM—10 μM), a specific NKCC1 activity blocker, with blue data representing control—no ALD. Cell lysates were collected in PBS buffer and cell fractions were separated using the cell signaling kit. Total lysate and membrane fractions were analyzed using western blot techniques, and significant increases in NKCC1 protein expression were observed with ALD and BUM, as compared to controls (n = 3). **b)** BUM treatment has no effect on NKCC1 gene expression, as shown by real-time RT-PCR (n = 3). **c)** Cells were treated with ALD in the presence of 1 mM TEA and increases in NKCC1 protein expression were observed for total lysate and membrane fraction (n = 3). (**d**) NKCC1 gene expression was not changed by TEA real-time RT-PCR methods (n = 3). β-actin was used as the control for total lysate, and total lysate concentration was used to achieve the equal loading for membrane fractions. Bar graph data are presented as mean ± SEM. Statistical Significance: **P < 0.01, ***P < 0.001, ****P < 0.0001.

Since ALD upregulated NKCC1 protein expression without affecting mRNA levels, we then tested if a similar change could be observed by directly blocking NKCC1 functional activity using a selective NKCC1 antagonist, bumetanide (BUM) during ALD treatment. There are reports that in vivo administration of BUM leads to a downregulation of NKCC1 protein levels^62,63^. In presence of BUM, NKCC1 protein expression was still found to be upregulated for both total lysate and membrane fractions, consistent with ALD treatment alone (Fig. 4a). There was also no change in gene expression (Fig. 4b). These data suggest that blocking NKCC1 activity does not prevent ALD induction of NKCC1 protein expression. ALD regulates both ion channels as well as ion co-transporters^7,64–66^. Ion channels, solute carriers and pumps move ions and other solutes in and out of cells, having a wide range of specificities, transport rates and biological processes. These functions are crucial for production and secretory processes, essential to intercellular communication^67^. We would therefore expect some crosstalk between NKCC1 and other potassium channels during ALD stimulation. Additional experiments were also done, combining ALD with tetraethyl ammonium (TEA—1 mM), a blocker for potassium channels. We tested ALD effects on NKKC1 expression in the presence of TEA. There was no effect on upregulation of NKCC1 protein expression in the presence of ALD when TEA was added (Fig. 4c), nor was an increase observed in NKCC1 gene expressions (Fig. 4d). These data indicated that there is no cross talk between NKCC1, and TEA-dependent potassium channels based with the induction of NKCC1 protein expression by ALD.

### ALD mineralocorticoid receptor and NKCC1 inhibitors affect ALD-induced whole-cell currents

**Figure 5:**
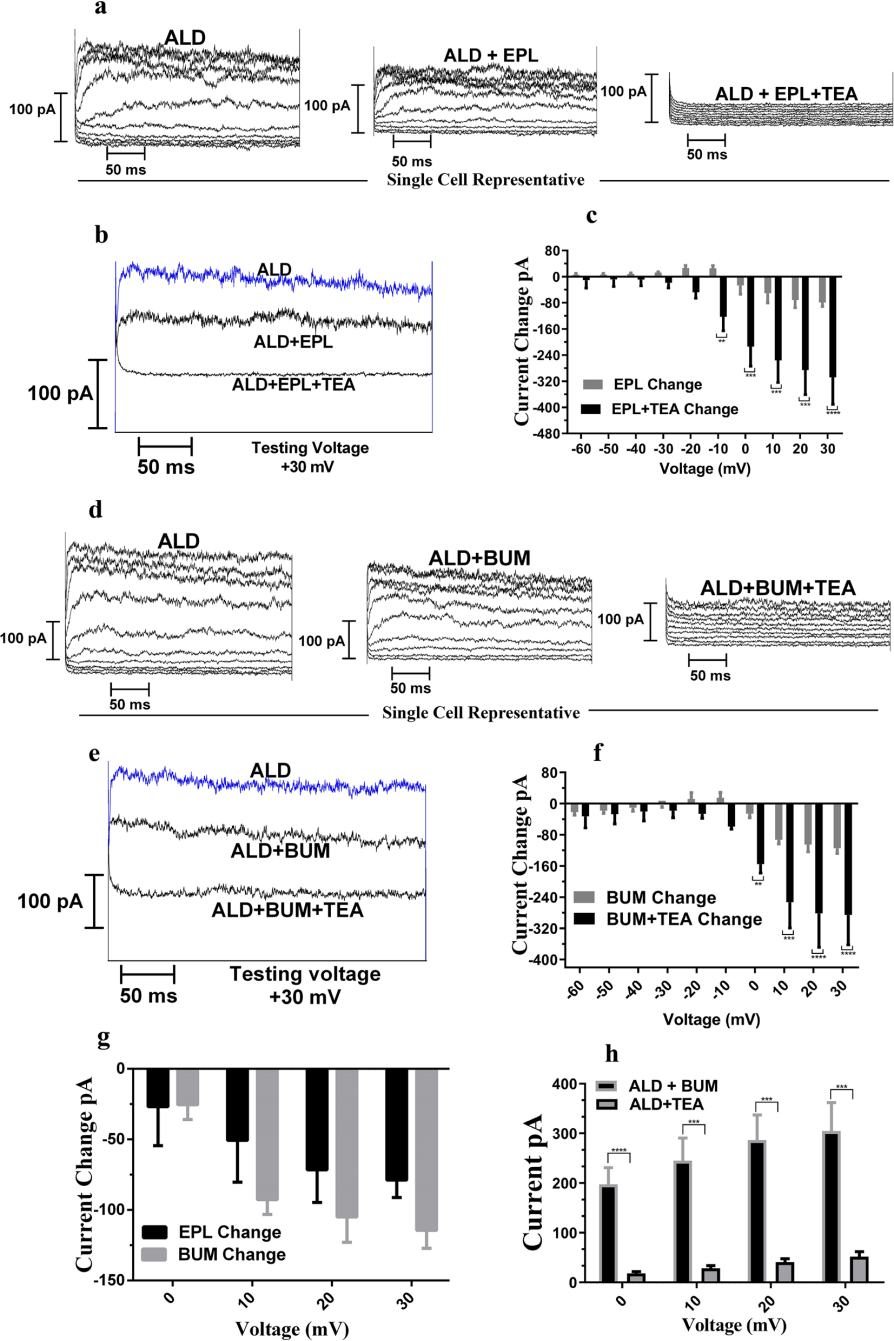
The NKCC1 inhibitor bumetanide, and mineralocorticoid receptor inhibitor EPL, alter ALD mediated increases in whole cell currents in SY5Y cells compared to ALD alone. (**a**) Whole-cell voltage clamp traces of a representative SH-SY5Y cell when ALD (1 μM) was applied (left) or ALD was applied together with 20 μM of EPL (middle) and or ALD was applied with EPL and TEA. The outward current was decreased with application of ALD with EPL, as compare to ALD alone and currents further reduced by application of ALD with EPL and TEA, indicating the involvement of outward potassium currents. Testing Potentials: −60 mV to + 30 mV, 10 mV increment; Holding Potential: −90 mV **b)** At a testing potential of + 30 mV a similar result is observed: a decrease in currents due to application of ADL in combination of EPL and EPL + TEA. (**c**) The decrease in mean currents with respect to ALD values (ALD vs. ALD + EPL & ALD vs ALD + EPL + TEA) is also prominent for positive holding potentials (n = 3). (**d**) Whole cell current of a representative SH-SY5Y cell when ALD (1 μM) was applied (left), ALD was applied along with 10 μM of BUM (middle) and ALD was applied with BUM & TEA. The outward current was decreased when ALD with BUM was applied and when ALD + BUM + TEA was applied, as compared to ALD alone. Testing Potentials: -60 mV to + 30 mV, 10 mV increment; Holding Potential: −90 mV (**e**) The decrease in currents due to application NKCC1 & TEA blockers are shown at testing voltage + 30 mV. (**f**) Similar to panel c, the mean decreases in currents with respect to ALD values due to application of BUM alone and BUM + TEA were more prominent at positive potentials (n = 3). (**g**) The comparison of mineralocorticoid receptor blocker and NKCC1 blocker current changes (with respect to ALD currents) shows that both blockers have similar levels of current reduction (n = 3). (**h**) The difference between currents of ALD minus outward currents in the presence of TEA and BUM indicates the involvement of NKCC1 in ALD-induced potassium currents. Statistical Significance: **P < 0.01, ***P < 0.001, ****P < 0.0001.

Currently, there are no reports on the effects of ALD on voltage-gated K^+^ currents. However, ALD has been shown to decrease transient outward currents in rat ventricular cardiomyocytes^68^. Potassium channels also help to maintain membrane voltages of ALD-sensitive distal nephrons, and play roles in ALD secretion and inner ear potassium circulation^29,66,69^. We hypothesized that ALD would regulate voltage-gated K^+^ currents with involvement of NKCC1 modulation linked to mineralocorticoid receptors. To determine that ALD-induced currents are linked to mineralocorticoid receptors, cells were perfused with ALD in the presence of EPL—an ALD mineralocorticoid receptors antagonist, and in the presence of EPL and TEA—a potassium channel blocker. We observed a decrease in ALD-induced currents when ALD was applied together with EPL, and a further decrease in currents were observed when ALD was applied with EPL + TEA (Fig. 5a). Current traces recorded at a + 30 mV testing potential (holding potential = – 90 mV) confirms the same (Fig. 5b). We also found that the addition of blockers (EPL or EPL + TEA), in the presence of ALD, decreases the ALD-induced whole cell currents, especially at positive testing potentials (Fig. 5c).

To determine the role of NKCC1 in ALD-elicited currents, ALD was applied in the presence of bumetanide (BUM)—a specific activity blocker for NKCC1, and BUM + TEA. The addition of BUM decreased the whole-cell currents, especially for positive testing potentials as seen in Fig. 5 d,e for a single cell, with further decreases in currents for addition of BUM and TEA together. Figure 5f shows that mean reduction in currents due to application of blockers (BUM and BUM + TEA) for 3 different cells, indicating the involvement of NKCC1 for modulating ALD-induced currents. The results suggest that the, NKCC1 blocker – BUM seems to be more comparable to the mineralocorticoid receptor blocker—EPL and there was no significant difference between application of EPL and BUM, the NKCC1 blocker (Fig. 5g) The difference in inhibition of currents between BUM and TEA is shown in Fig. 5h, confirming that the decrease in current was more prominent for positive testing potentials. These results are consistent with our findings presented above, where it was shown that BUM and EPL could not block the induction of NKCC1 cell surface protein expression induced by ALD (Fig. 4). This supports the concept that a mechanism where ALD increases voltage-gated K^+^ currents involving NKCC1 modulation via mineralocorticoid receptors. ALD induced currents were sensitive to TEA – a specific potassium blocker, as application of 1 mM TEA suppressed the currents elicited by ALD (Figs. 1,5). ALD-induced currents were dose independent because experiments with 0.1 µM and 10 µM change the currents the same amount as 1 µM (data not shown). The use of specific K + antagonists confirm that ALD induced potassium up-regulation was mediated by mineralocorticoid receptors, as EPL was an effective antagonist as shown in Fig. 5a–c and involves NKCC1 co-transporter (BUM blocker—Fig. 5d–f). There may be an indirect mechanism for the observed reduction in K^+^ currents as the ALD-mineralocorticoid receptor interactions may initiate post-transcriptional gene regulation via SGK1, CNK3 which can lead to modification of ion channels. Similarly, the effects of BUM could also be through indirect rather that direct binding to potassium channels. For instance, Wang and co-workers studied the effects of bumetanide on cellular uptake of gentamicin in kidney cells. They found that BUM induced an outward currents and activated K^+^ conductance. The indirect underlying hypothesis was that loop diuretic induced currents were indirectly activated by increasing the cytosolic calcium and mediated by calcium sensitive TRPV channels ^70^. Also, cell volume regulation, a fundamental activity of cells, activates various ion channels pathways like K^+^ and Cl^−^ coupled with different co-transporters like K^+^–Cl^−^, Na^+^/H^+^ exchange and NKCC1**.** There are reports that cell volume is regulated by bumetanide sensitive ion-transports and involves potassium flux^71–73^. Further investigation is needed to decipher detailed pathways involved.

## Discussion

Previously we reported that long-term systemic treatment with ALD dramatically improves hearing function and reduces expression of cochlear apoptotic biomarkers in aging mice^39,40^. In the current study we examined the cellular mechanisms that may underlie these biotherapeutic effects of chronic ALD administration. We found that applying ALD to neuronal cells in vitro increased NKCC1 whole lysate and membrane portion expression levels, however NKCC1 mRNA expression was not upregulated, indicating that the modulation of NKCC1 function by ALD is a post-transcriptional modification. These findings are consistent with our earlier report^4^ that ALD treatment of HT-29 cells lead to increase in NKCC1 protein expression, mediated by mineralocorticoid receptors, without affecting mRNA levels. The upregulation of NKCC1 expression via ALD treatment increased the outward potassium currents in SH-5HSY cell lines with confirmed NKCC1 gene and protein expression levels. Moreover, we observed ALD-stimulated potassium currents were sensitive to BUM – a NKCC1 blocker and EPL – a mineralocorticoid receptor blocker; indicating the involvement of NKCC1 mediated by the ALD-receptor complex. Taken together, these findings implicate NKCC1 as an important ion channel transporter for modulation of voltage-gated potassium currents by ALD and may play a role ion channel dysfunction in the aging ear. The similarity of the inhibitory actions between BUM and TEA, as well no significant differences between EPL and BUM actions for ALD-induced currents, were other indications that NKCC1 co-transporters affect potassium currents.

In tissues which express mineralocorticoid receptors, ALD modulates the expression of membrane targets such as the subunits of the epithelial Na^+^ channel, in combination with important signaling intermediates, such as serum and glucocorticoid-regulated kinase-1. In addition, the rapid ‘non-genomic’ activation of protein kinases and secondary messenger signaling cascades has also been detected in ALD-sensitive tissues of the nephron, distal colon and cardiovascular system. These rapid cell membrane actions have been linked to mineralocorticoid receptors or to an as yet unidentified, membrane-associated ALD receptor^74^. Our observations indicate that the time course in which ALD modulates NKCC1 protein expression is rapid, pointing to non-genomic mechanisms. These actions likely involve a cascade reaction from receptors, and signals to membrane targets, implicating a signal pathway involving mineralocorticoid stimulation. In addition, although ALD mediates Na^+^ retention and K^+^ excretion via a genomic pathway through cytosolic mineralocorticoid receptors, which can alter transcription^75^, our study indicates that the rapid action of ALD on NKCC1 protein expression (both in whole cell lysate and membrane fractions) and voltage-gated potassium currents are associated with the activation of mineralocorticoid receptors. This suggests that the classic cytosolic mineralocorticoid receptor can also be involved in mediating non-genomic ALD signaling.

We previously demonstrated that ALD up-regulation of NKCC1 protein expression exerts its effects via prevention of post-translational ubiquitination, e.g., reduces proteasome-dependent degradation of the NKCC1 protein^4^. In the present study, we also observed that NKCC1 membrane protein upregulation was associated with whole cell lysate NKCC1 protein expression and voltage-gated current enhancements. Consistent with the finding that NKCC1 is a basolateral membrane protein, Rinehart et al.^76^ observed that the upregulation of its cell surface distribution would increase its activity. A similar mechanism may underlie findings of the current study showing an increased NKCC1 membrane portion accompanies voltage-gated potassium current increases induced by ALD.

Regulation of membrane bound NKCC1 involves acute changes in the cell surface expression of NKCC1 via two different mechanisms involving dynamic changes in membrane recycling. The first is via the cAMP pathway, where D’Andrea et al. suggested that cAMP may induce the surface recruitment of membrane proteins that form a regulatory complex with NKCC1^77^ and it has been shown under a variety of experimental circumstances that NKCC1 activity is strongly affected by the F-actin cytoskeleton^78–83^. Second, Protein kinase C enhances the endocytic retrieval of NKCC1 from the plasma membrane and may be a mechanism for short-term downregulation of epithelial secretory capacity^80,83–85^. Bouyer and colleagues have shown that NKCC1 is endocytosed by a clathrin-mediated pathway during Protein kinase C activation by phorbol 12 myristate 13-acetate^86^. Similar mechanisms could account for the rapid upregulation of NKCC1 membrane protein because ALD involves in pathways of cAMP and Protein kinase C activation as the rapid action of ALD on NKCC1 may exert an important role in the alternation of the cell membrane voltage-gated potassium currents.

NKCC1 is highly expressed in neurons, e.g. inner ear spiral ganglion (cochlear) and vestibular ganglia^23^. Although the role of the cotransporter in these specific neurons is not clear, considering the present study, one role of NKCC1 in the cochlea appears to be mediated via ubiquitination. As in epithelia, the Na^+^/K^+^ pump helps provide forces for K^+^ and Cl^−^ influx through NKCC1 channels. While Na^+^ and Cl^−^ are subsequently recycled respectively by the Na^+^ /K^+^ pump and chloride channels—CLC-K, K^+^ is secreted into the scala media by NKCC1 and a complex of K^+^ channels, specifically KCNQ1 and KCNE1^87–89^. It is tempting to speculate that the cotransporter in neurons might be participating in cross talk with potassium channels, since our study discovered the presence of overlapping inhibition actions between BUM and TEA for ALD-induced potassium currents in a neuronal cell line. Also, these changes are linked to mineralocorticoid receptors as ALD stimulation for NKCC1 proteins and K^+^ currents were sensitive to receptor blockers (Fig. 5).

Bursts of neural network activity are associated with transient increases in extracellular K^+^ levels. This excess K^+^ is partially removed from the extracellular space by a combination mechanism proposed to involve Kir4.1-mediated spatial buffering, the Na^+^/K^+^/2Cl^−^ cotransporter (NKCC1), and/or Na^+^/K^+^-ATPase activity. However, the individual contributions of these ion transporters to [K^+^] management are controversial. For example, a study in rat hippocampal slices showed that inhibition of NKCC1 failed to affect the rate of K^+^ removal from extracellular space, while Kir4.1 initiated its spatial buffering only during a local [K^+^] increase. Inhibition of the different isoforms of Na + /K + -ATPase reduced post-stimulus clearance of K^+^ transients. Thus, NKCC1 played no role in activity-induced extracellular K^+^ recovery in native hippocampal tissue while Kir4.1 and Na^+^/K^+^-ATPase served temporally distinct roles^90^. Based on this information, we cannot exclude the possible involvement of voltage-gated potassium channels in the ALD rapid actions mediated by NKCC1. Further investigation is needed to understand how NKCC1 influences potassium channels by changing intracellular concentrations and/or cell volume. However, we provide solid evidence that these changes are mediated by ALD-sensitive mineralocorticoid receptors.

## Summary and conclusion

The increase in voltage-gated potassium currents following ALD application can be explain by NKCC1 upregulation in the cell membrane, mediated by mineralocorticoid receptors. The similarity between the results observed with BUM and TEA on inhibition of ALD induced voltage-gated K^+^ currents suggests that the cross talk and interplay between NKCC1 and K^+^ channels, and their mechanisms need further clarification. Overall, NKCC1 plays a key role in various physiological systems including the cochlea, and its age-related declines. Previously, we have shown that long-term systemic treatment with ALD can prevent key aspects of ARHL in aging mice, indicating that ALD could potentially be a therapeutic agent for ARHL clinically^40^. Also, long-term treatment with ALD increased the serum ALD levels in treated animals as compared to control animals, which improved survival of spiral ganglion neurons through upregulation of mineralocorticoid receptors^39^. The present article along with our previous reports elucidate the possible molecular mechanisms that ALD exerts in auditory peripheral function following ALD treatments. More specifically, the results presented in this report are proof-of-concept that ALD upregulates NKCC1 in cell membrane fractions and for potassium currents. These effects were mediated by mineralocorticoid receptors and the NKCC1 co-transporter. To quantify the role of NKCC1 activity in ALD-elicited currents in cochlear cells, including hair cells, our future work will be focused on performing electrophysiological measurement with cochlear explants treated with ALD in combination with other potentially therapeutic compounds. To further confirm new discoveries here, a key, challenging experiment would be to perform siRNA silencing of NKCC1 in cochlear explants and study the ALD effects on voltage-gated K^+^ currents. Also, it would be useful to study various NKCC1 and potassium current blocker effects alone, as well combining other techniques like CRISPR-Cas 9, on the ionic currents and protein/gene expressions to decipher further details. This will help elucidate the cellular and molecular mechanisms by which ALD modulates NKCC1 and K^+^ channel functions, to improve hearing, and treat acquired forms of hearing loss such as ARHL.
